# Prevalence of resistant hypertension and associated factors for blood pressure control status with optimal medical therapy using Korean ambulatory blood pressure monitoring registry data

**DOI:** 10.1186/s40885-016-0045-x

**Published:** 2016-01-27

**Authors:** Sung Il Choi, Soon Kil Kim, Sungha Park, Ju Han Kim, Sang Hyun Ihm, Gwang-il Kim, Woo Shik Kim, Wook Bum Pyun, Yu-Mi Kim, Jinho Shin

**Affiliations:** 1 Cardiology division, Department of Internal Medicine, Hanyang University, College of Medicine, 222 Wangsimni-ro Sungdong-Ku, Seoul, #133-792 South Korea; 2 Department of Internal Medicine, Yonsei University, School of Medicine, Seoul, Korea; 3 Department of Internal Medicine, Chonnam University, School of Medicine, GwangJu, Korea; 4 Department of Internal Medicine, Catholic University, College of Medicine, Bucheon, Korea; 5 Department of Internal Medicine, Seoul National University, School of Medicine, Bundang, Korea; 6 Department of Internal Medicine, Kyung Hee University, School of Medicine, Seoul, Korea; 7 Department of Internal Medicine, Ewha Womans University, School of Medicine, Seoul, Korea; 8 Department of Preventive Medicine, Dong-A University College of Medicine, Busan, Korea

**Keywords:** Hypertension Resistant to Conventional Therapy, Ambulatory blood pressure monitoring, Masked hypertension, White-coat hypertension, Hypertension

## Abstract

**Background:**

Resistant hypertension (RH) may be one of the cause of the plateau in improving the control rate in hypertension (HT) management. The misdiagnosis of RH by clinic blood pressure (BP) is important clinical problem. Aim of the study were to investigate the prevalence of RH by ambulatory blood pressure monitoring (ABPM) and the factor associated with control status of ambulatory BPs.

**Methods:**

For 1230 subjects taking one or more antihypertensive medication (AHM) enrolled in the Korean Ambulatory Blood Pressure Monitoring (Kor-ABP) registry, the prevalence of RH was calculated which was defined as uncontrolled BP by three AHM classes including diuretic or BP in need of four or more AHM classes. The prevalence determined by clinic versus ambulatory BP was compared.

**Results:**

The age was 59.3 ± 12.5 years, and 44.3 % were female (*n* = 1230). Among them 72 subjects were taking three AHM drugs including diuretics and 105 subjects were taking four or more AHM classes. With uncontrolled daytime ambulatory BP in 41 among 72 subjects, prevalence of RH was 11.9 % (146/1230). By using nighttime BP criteria, there was significant difference in the prevalence of RH for clinic versus nighttime BP (146/177 vs. 159/177, *p* = 0.0124). For control status of daytime BP, masked uncontrolled BP was 16.9 % and controlled BP with white-coat effect was 14.1 %. For nighttime BP control status, odd ratios for smoking (0.624), drinking (1.512), coronary artery disease (0.604), calcium antagonist (1.705), and loop diuretics (0.454) were all significant.

**Conclusion:**

The prevalence itself was 11.9 % by daytime BP and it was significantly higher when using nighttime BP criteria. Control status of daytime BP was misclassified in 31.0 %. Smoking, drinking, coronary artery disease, calcium antagonist, and loop diuretics were associated with nighttime BP control status.

## Background

Resistant hypertension (RH) is defined as the hypertension (HT) which cannot be controlled below the target blood pressure even if treated with three or more antihypertensive medications (AHM) classes one of which was diuretics or HT which requires four or more AHM classes regardless whether the blood pressure was controlled or not [1]. In recent years, the trend in the control rate of HT in the treated patient has reach a plateau just below 70 % in Korean population [2]. Because the control of HT is essential component in preventing cardiovascular events, the obstacle against improving the control rate of HT is a very important clinical issue and the epidemiologic study is also very important to provide a basis for setting up management strategy of RH.

As shown in the recent clinical trial, the learning from the misdiagnosis of RH using only clinic BP made out-of-office BP measurement, preferably ABPM, as the essential component of the evaluation before adopting the new therapeutic modality [3]. So far, there are few studies for the prevalence of the RH by using ABPM in Korean situation. And the amount of the diagnostic error in using clinic BP to diagnose RH was either unknown in general. And as long as the accurate diagnosis of RH using ABPM is concerned, there are few report regarding the demographic or clinical factors related to the RH.

So the aim of the present study is, firstly, to identify the true prevalence of RH by analyzing the multicenter ABPM registry data in Korea (Kor-ABP registry) and to identify the diagnostic errors of clinic BP in the diagnosis of RH. Secondly, it is to identify the demographic and clinical factor related to the treatment resistant condition in the subject treated three or more AHM classes.

## Methods

### Subjects

Among 3766 subjects who were enrolled for Kor-ABP registry during the period from 1st August, 2009 to the end of 2012, 1242 subjects with relevant clinical information and who were treated with at least one AHM were selected for the study. The subject was registered by 27 referral hospitals as described in the previous study [4]. The exclusion criteria was estimated glomerular filtration rate (eGFR) calculated by CKD-EPI equation [5] less than 30 ml/min/1.73m2 and finally 1230 subjects was analyzed for the study.

### Clinical and laboratory variables

Information included from the medical records covered: age, gender, height, weight, abdominal circumference, presence of diabetes mellitus (DM), hyperlipidemia, presence of clinical cardiovascular disease, time of diagnosis, mode of treatment, and prescribed medications.

And the questionnaire for each subjects included: smoking status, alcohol intake, extent of physical exercise, family history of HT and premature cardiovascular death, past medical history; HT, DM, hyperlipidemia, stroke, coronary artery disease (CAD), and heart failure.

The collected information for the AHM was the commercial names of the drugs and dosing at the time of enrollment.

### Clinic BP and ABPM data

Clinic BP was measured using an A&D UA-767, which passed European Society of Hypertension protocol and International Protocol [6]. Clinic BP was defined as the average BP of two measurements one minute apart, with 5 min’ rest before the first measurement.

ABPM data were gathered as the form of raw data files uploaded to the study website. The device used in the study institute was all recommended as summarized in the website http://www.dableducational.org or passed the recommended validation protocols [7]. The raw data of ABPM were regarded as valid only when at least 14 readings of the awake blood pressure from 8AM to 9PM were available after omitting erroneous readings according to the following criteria [8]:

1. pulse rate above 120 beats per minute
2. systolic or diastolic blood pressure more than 25 mmHg above or below the previous and subsequent readings
3. pulse pressure below 15 mmHg [9].

### Definition of RH and groups according to the control status of BP

The definition of the resistant hypertension was uncontrolled BP even if using three drug classes one of which included diuretics or the BP status requiring treatment using four or more drugs regardless of the control status [1]. The number of drug used was counted only when the dosage of the each drug was >50 % of the maximum recommended or approved dose for hypertension [10]. For example, for Angiotensin converting enzyme inhibitor (ACEI), ramipril 5mg or perindopril 4mg were minimal dosage needed for optimal treatment. Similarly, for example, losartan 50 mg, valsartan 80mg, amlodipine 5mg, diltiazem 90 mg, bisoprolol 2.5 mg, carvedilol 12.5 mg, nebivolol 2.5 mg, dihydrochlorthiazide 12.5 mg, furosemide 20mg, torsemide 5mg, indapamide 2.5mg spironolacton 12.5mg were the minimal required dosage.

BP was categorized as controlled and uncontrolled status according to the various criteria. By clinic BP criterion, the BP lowered below 140/90 mmHg was regarded as controlled status. As for daytime, nighttime, and 24 h BPs, the BPs lowered below 135/85 mmHg, 120/70 mmHg, and 130/80 mmHg were regarded as controlled status, respectively [2, 11].

For the subjects taking three or more AHM including diuretics, according to the control status defined by various criteria, the subject with controlled BP status was grouped as responsive group, whereas the subject with uncontrolled BP status was grouped as the resistant group.

### Definitions of the clinical conditions

Dyslipidemia was defined as the total cholesterol ≥ 240 mg/dL, triglyceride ≥ 150 mg/dL, HDL < 45/50 mg/dL in male and female, respectively, or the use of statin. The diabetes was defined as fasting blood glucose ≥ 126 mg/dL, hemoglobin A1C > 6.5 %, or the use of antidiabetic medication. The chronic kidney disease (CKD) was defined as eGFR calculated by CKD-EPI equation between 30 and 60 ml/min/1,73m2 [5]. Smoking was defined as current smoker and drinking was defined as current drinking by the questionnaire. Regular physical exercise was defined as the three or more times of structured exercise per week. Metabolic syndrome (Mets) was defined when the subject had three or more components of impaired fasting blood glucose or diabetes mellitus, obesity defined by increased abdominal circumference (≥90 cm in male; ≥ 80 cm in female) or BMI ≥ 25 kg/m2, triglyceride level ≥ 150 mg/dL, low HDL level, and blood pressure ≥ 130/85 mmHg or a history of hypertension [12]. Global cardiovascular risk (GCR) profiles were determined according to the 2013 Korean Society of Hypertension Guideline [2].

The study protocol was approved by the clinical research ethics committees in all hospitals involved in the study.

### Statistical analyses

All data were expressed as mean ± standard deviation. The statistical significance of the differences in mean values was evaluated using student t-test or analysis of variance (ANOVA). The chi square test and Fisher’s exact test were used to ascertain the statistical significance of the categorical difference between groups. The differences in diagnostic category between clinic versus ambulatory BPs were evaluated using McNemar’s test. Test for inter-rater reliability using the kappa test statistics with 95 % confidence interval were performed to determine the consistency of the two methods of measuring blood pressure.

Multiple logistic regression analysis was performed to examine the association between resistant group defined by various BP criteria and the clinical factors. The independent variable included age, sex, Mets, family history of HTN, physical activity, drinking and smoking status, DM, CKD, history of stroke, CAD, heart failure, the use of ACEI or angiotensin receptor blocker (ARB) as renin angiotensin system (RAS) blockade, beta blocker (BB), calcium channel blocker (CCB), and loop diuretics, and the use of optimal dosage. Optimal dosage was defined as the case all of the drug dosage should be ≥ 50 % of the maximum recommended dosage. Statistical significance was defined by a confidence interval of 95% and *p* < 0.05. All data processing and analysis were performed using SAS 9.4 (SAS Institute Inc., Cary, NC, USA).

## Results

### General characteristics of the study subjects

The age was 59.3 ± 12.5 years, and 44.3 % were female (*n* = 1230). Body mass index was 25.2 ± 3.7 in male and 24.9 ± 3.6 in female (*p* = 0.0984). Smoking and drinking were more frequent in male than female (11.8 % vs 0.8%, *p* <0.0001 and 29.5% vs 7.2%, *p* < 0.0001). Family history of HT was present in 46.0 %. High GCR was 83.3 % and coronary artery disease, stroke and heart failure were 25.7 %, 12.6 %, and 4.8 %, respectively. CKD was observed in 10.6 %.

ARBs prescribed were irbesartan (23.1 %), olmesartan (19.0% ), telmisartan (15.7 %), valsartan (15.7 %), losartan (12.4 %), candesartan (8.3 %), fimasartan (1.7 %), and eprosartan (4.1 %). Fixed drug combination was prescribed in 48.7 % for ARB and diuretics. The majority of ACEI was perindopril (70.5 %). BBs prescribed were bisoprolol (39.3 %), carvediolol(29.9 %), nebivolol(12.0 %), atenolol(12.0 %), and etc(2.5 %). CCBs prescribed were amlodipine (49.1 %), slow release form of nifedipine (15.7 %), diltiazem (8.3%), and etc (26.8 %). Diuretics prescribed were dihydrochlorthiazide (65.9 %), loop diuretics (17.4 %), indapamide (7.5 %), and spironolactone (6.6 %).

As shown in Table 1, among 1230 subjects, 72 subjects took three drug classes one of which included diuretics and 105 subjects took four or more drug classes. As for clinical BP, BP in 41 among 72 subjects were uncontrolled.

**Table 1:** The uses of antihypertensive medications in 1230 study subjects according to the optimal disage criteria

	Count by any dosage	Count by 50 % or more of recommended dose	p*
Angiotensin receptor blocker	714 (57.9 %)	705 (57.2 %)	0
ACE inhibitor	166 (13.5 %)	157 (12.7 %)	0
Beta blocker	530 (43.0 %)	456 (37.0 %)	< 0.0001
Calcium antagonist	732 (59.4 %)	727 (59.0 %)	0.03
Dihydrochlorthiazide	259 (21.0 %)	235 (19.1 %)	<0.0001
Indapamide	19 (1.5 %)	19 (1.5 %)	0
Loop diuretics	49 (4.0 %)	47 (3.8 %)	0.16
Spironolactone	20 (1.6 %)	20 (1.6 %)	0
Categories of antihypertensive drug regimen			
3 drugs including diuretics	72 (5.9 %)	83 (6.7 %)	<0.0001
4 drugs	105 (8.5%)	83 (6.7 %)	
other 1 or more drugs	1053(85.6 %)	1064(86.5 %)	

*ACE* angiotensin converting enzyme, * p value for McNemar chi square test

When the dosage of each drug ≥ 50 % of the maximum recommended or approved dose for hypertension was considered effective treatment, 83 subjects took three drugs including diuretics and 83 subjects took four or more drug classes and the categorization was differed (McNemar *p* < 0.0001). As for clinic BP, BP of 42 among 83 subjects taking three drugs including diuretics were uncontrolled.

### Prevalence of RH

BP in 41 (56.9 %), 41 (56.9 %), 54(75.0 %), and 47(65.2 %) subjects of the 72 subject taking three AHMs including diuretics were not controlled by clinic, daytime, nighttime, and 24 h BP criteria (Table 2). Considering 105 subjects taking four or more drugs, among the HT subject taking at least one AHM, the prevalence of RH was 11.9 % (146/1230), 11.9 % (146/1230), 12.9 % (159/1230), and 12.3 % (152/1230) according to the criteria by clinic, daytime, nighttime, and 24 h BPs, respectively. But Among the subjects taking three or more AHMs, the prevalence of the RH was 82.4 % (146/177), 82.4 % (146/177), 89.8 % (159/177), and 85.9 % (152/177) according to the criteria by clinic, daytime, nighttime, and 24 h BPs, respectively. Because all subjects taking four or more drugs are classified as RH, the prevalence can be sensitive to the control status of the 72 subject taking three drugs including diuretics.

**Table 2:** Agreement between clinic blood pressure and ambulatory blood pressure to classify resistant hypertension

		Clinic BP		p*	Kappa
		Non-RH	RH		(95% confidence interval)
Daytime BP	Non-RH	19 (10.7 %)	12 (6.8 %)		0.53
	RH	12 (6.8 %)	134 (75.7 %)	1	(0.3658 ~ 0.6956)
Nighttime BP	Non-RH	11 (6.2 %)	7 (4.0 %)		0.37
	RH	20 (11.3 %)	139 (78.5 %)	0.01	(0.1813 ~ 0.5539)
24 h BP	Non-RH	17 (9.6 %)	8 (10.2 %)		0.53
	RH	14 (7.9%)	138 (78.0%)	0.2	(0.3638 ~ 0.7049)

*BP* blood pressure, *RH* resistant hypertension defined by uncontrolled BP by each criteron or BP control needing four or more antihypertensive medications. *,p for McNemar test

For the subject taking optimal dosage, the prevalence of RH was 10.1 % (125/1230), 10.4 % (129/1230), 11.2 % (139/1230), 10.8 % (134/1230) according to the criteria by clinic, daytime, nighttime, and 24 h BPs, respectively.

### Agreement of clinic BP and ambulatory BP for the diagnosis of RH

According to the definition of RH, the subject with uncontrolled BP among 72 subjects taking three AHMs including diuretics and all of the subjects taking four or more AHMs (*n* = 105) were classified as RH. As shown in Table 3, the diagnosis rate was significantly different when using nighttime BP criterion even though kappa index was similar and poorly agreed.

**Table 3:** Agreement between clinic blood pressure and ambulatory blood pressure in the control status in the subject taking three or more drugs

		Clinic BP (mmHg)		p*	Kappa
		<140/90	> = 140/90		(95 % confidence interval)
Daytime BP (mmHg)	<135/85	30 (16.9 %)	25 (14.1 %)		0.29
	> = 135/85	30 (16.9 %)	92 (52.1 %)	0.5	(0.1443 ~ 0.4402)
Nighttime BP (mmHg)	<120/70	19 (10.7 %)	18 (10.2 %)		0.18
	> = 120/70	41 (23.2)	99 (55.9 %)	0	(0.0328 ~ 0.3264)
24 h BP (mmHg)	<130/80	27 (15.3 %)	18 (10.2 %)		0.32
	> = 130/80	33 (18.6 %)	99 (55.9 %)	0.04	(0.1680 ~ 0.4627)

*BP* blood pressure, *, p for McNemar test

### Agreement of the control status in the subject taking three or more AHMs

As shown in Table 3, the control status defined by nighttime and 24 h BP criteria were significantly different compared to the status defined by clinic BP criterion. At least more than 50 % of the subject taking three or more AHMs showed consistently uncontrolled BP regardless of the kind of the criteria applied. Among 177 subjects taking three or more drugs, 117(66.1 %), 122(68.9 %), 140(79.1 %), and 132(74.5 %) were uncontrolled by clinic, daytime, nighttime, 24 h BP criteria. The kappa statistics suggested poor agreement showing that 31.0% (*n* = 55), 33.4 % (*n* = 59), and 28.8 % (*n* = 51) were misclassified as uncontrolled BP due to white-coat effect or misclassified as controlled BP due to masking by the clinic BP. For control status of daytime BP, masked uncontrolled BP was 16.9 % and controlled BP with white-coat effect was 14.1 %. Masked uncontrolled HT was more frequently observed when using nighttime BP criterion than daytime BP criterion (16.9 % vs. 23.1 %, *p* = 0.0343). Because daytime BPs in 31 subjects were controlled as shown in Table 2, among 105 subjects taking four or more drugs, 91 subjects (86.6 %) as shown in Table 3 were uncontrolled in daytime BP.

### Characteristics of the controlled versus uncontrolled BP groups

As shown in Table 4, when the criteria for control status by daytime ambulatory BP, there was no significant difference between controlled BP group and uncontrolled BP group in terms of demographic and clinical profiles except for BP levels. Significantly higher CAD and heart failure were observed in controlled BP group than uncontrolled BP group. In terms of AHMs, in uncontrolled BP group, BB and CCB were more frequently prescribed whereas loop diuretics were more frequently prescribed in controlled BP group.

**Table 4:** The comparison according to control status by daytime blood pressure of 135/85 mmHg or greater in response to three or more antihypertensive medications including diuretics

	Controlled BP group	Uncontrolled BP group	*p*
	(*N* = 55)	(*N* = 122)	
Age (yr)	61.6 ± 11.2	61.2 ± 10.4	0.84
Body mass index (Kg/m2)	25.7 ± 4.1	25.6 ± 3.3	0.83
Abdominal circumference (cm)	93.8 ± 10.9	91.9 ± 9.3	0.28
Clinic systolic BP (mmHg)	135.1 ± 18.2	153.8 ± 24.1	<0.0001
Clinic diastolic BP (mmHg)	80.2 ± 12.3	91.5 ± 16.1	<0.0001
Heart rate (beats per minute)	73.7 ± 14.4	75.2 ± 14.8	0.58
Potassium (mEq/L)	4 ± 0.4	4.1 ± 0.5	0.37
Creatinine (mg/dL)	1 ± 0.3	0.9 ± 0.2	0.14
Cholesterol (mg/dL)	171.4 ± 40.7	182.9 ± 44.9	0.17
Fasting blood glucose (mg/dL)	114.2 ± 34	109.6 ± 34.6	0.53
Hemoglobin A1C (%)	6.6 ± 1.9	6.3 ± 1.1	0.37
Triglyceride (mg/dL)	128.3 ± 54.2	148.4 ± 81	0.19
High density lipoprotein (mg/dL)	46.7 ± 13.1	45.8 ± 10	0.67
Daytime systolic BP (mmHg)	121.8 ± 7.9	149.6 ± 15.2	<0.0001
Daytime diastolic BP (mmHg)	74.8 ± 5.3	90.9 ± 11.7	<0.0001
Daytime heart rate (bpm)	71.4 ± 9.6	73.9 ± 10.2	0.14
Nighttime systolic BP (mmHg)	115.5 ± 14	138.5 ± 20.5	<0.0001
Nighttime diastolic BP (mmHg)	71 ± 9.7	83.6 ± 15.4	<0.0001
Nighttime heat rate (bpm)	61.8 ± 10.3	64.8 ± 11.3	0.11
24 h systolic BP (mmHg)	121 ± 8.9	146.9 ± 15.3	<0.0001
24 h diastolic BP (mmHg)	73.9 ± 5.8	89.5 ± 12.7	<0.0001
24 h heart rate (bpm)	68.6 ± 9.3	71.3 ± 10	0.11
Smoking	14.6 %	11.5 %	0.57
Drinking	29.1 %	32.8 %	0.62
Regular physical activity	43.6 %	36.9 %	0.39
Family history of SCD	3.6 %	6.6 %	0.35
Family history of hypertension	36.4 %	46.7 %	0.2
Metabolic syndrome	47.3 %	44.3 %	0.71
High global CV risk	89.1 %	84.3 %	0.4
Cardiovascular disease history	60.0 %	45.1 %	0.07
Coronary artery disease	36.4 %	26.5 %	0.18
Stroke	14.8 %	9.0 %	0.25
Heart failure	18.5 %	5.8 %	0.01
Antihypertensive medication			
ACE inhibitor	14.5 %	22.1 %	0.24
Angiotensin receptor blocker	69.1 %	78.7 %	0.17
Beta blocker	56.4 %	74.6 %	0.02
Calcium antagonist	50.9 %	69.7 %	0.02
Diuretics	100.0 %	99.2 %	0.5
Thiazide	70.9 %	78.5 %	0.27
Indapamide	7.3 %	5.8 %	0.71
Spironolactone	5.5 %	6.6 %	0.77
Loop diuretics	21.8 %	10.7 %	0.05
Alpha blocker	1.8 %	2.5 %	0.41
Vasodilator	0.0 %	2.5 %	0.55
Aspirin	50.1 %	54.1 %	0.76
Statin	40.0 %	40.9 %	0.9

*ACE* angitensin converting enzyme, *BP* blood pressure, *CV* cardiovascular, *SCD* sudden cardiac death

### The factors related to control status

**Fig. 1:**
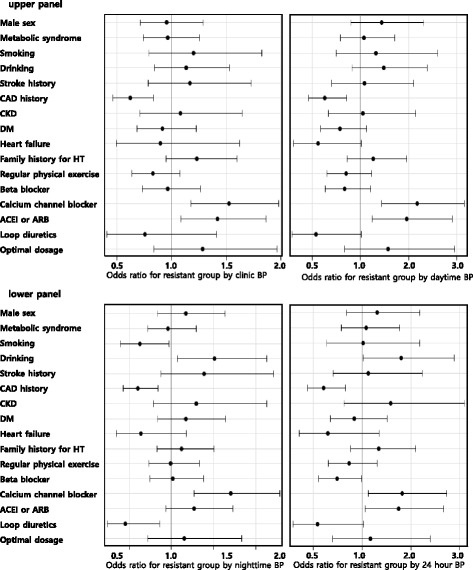
Demographic and clinical factors related to uncontrolled BP group when treated with three or more antihypertensive medications including diuretics. In the upper panel, CAD history, the use of calcium channel blocker or the use of ACEI/ARB were significant factor for uncontrolled BP group. In the lower left panel, additionally smoking, drinking, and loop diuretics were significantly related factor for uncontrolled BP group defined by nighttime BP ≥ 120/70 mmHg. In the lower right panel, CAD history, the use of calcium channel blocker or the use of ACEI/ARB were associated with uncontrolled BP group defined by 24 h BP ≥ 130/80 mmHg. ACEI, angiotensin converting enzyme inhibitor; ARB, angiotensin receptor blocker; BP, blood pressure ; CAD, coronary artery disease; CKD, chronic kidney disease; DM, diabetes mellitus; HT, hypertension

As shown in Table 5 and Fig. 1, smoking was related to controlled BP group for nighttime BP whereas drinking was related to the uncontrolled BP group for nighttime and 24 h BPs. History of CAD is associated with controlled BP group consistently regardless of the criteria applied. Heart failure was related to controlled BP group for daytime BP. Regarding the class of AHM, CCB was consistently related to the uncontrolled BP group regardless of the criteria applied. Specifically for nighttime BP, CCB was the only AHM class related to uncontrolled BP group. ACEI or ARB was also related to uncontrolled BP group by any criteria except for the nighttime BP. Interestingly, use of loop diuretics was associated with controlled BP group for nighttime BP.

**Table 5:** Multiple logistic regression analysis and odds ratios for the factors associated with the resistant group to antihypertensive theraphy using 3 or more antihypertensive medications including diuretics

	Clinic BP	Daytime BP	Nighttime BP	24 h BP
	^3^ 140/90 mmHg	^3^ 135/85 mmHg	^3^ 120/70 mmHg	^3^ 130/80 mmHg
Age	0.989[0.978 ~ 1.001]	0.992[0.98 ~ 1.004]	0.996[0.983 ~ 1.009]	0.992[0.98 ~ 1.005]
Male sex	0.956[0.71 ~ 1.288]	1.221[0.901 ~ 1.655]	1.171[0.836 ~ 1.639]	1.157[0.84 ~ 1.594]
Metabolic syndrome	0.965[0.743 ~ 1.253]	1.037[0.793 ~ 1.357]	0.963[0.717 ~ 1.294]	1.043[0.786 ~ 1.385]
Smoking	1.202[0.791 ~ 1.827]	1.161[0.75 ~ 1.797]	0.624[0.399 ~ 0.976]	1.008[0.639 ~ 1.59]
Drinking	1.135[0.843 ~ 1.528]	1.242[0.913 ~ 1.688]	1.512[1.072 ~ 2.133]	1.4[1.01 ~ 1.942]
Regular physical exercise	0.826[0.636 ~ 1.073]	0.855[0.654 ~ 1.119]	0.99[0.735 ~ 1.335]	0.869[0.654 ~ 1.153]
Family history of hypertension	1.231[0.947 ~ 1.6]	1.131[0.865 ~ 1.48]	1.121[0.834 ~ 1.507]	1.169[0.88 ~ 1.552]
Diabetes mellitus	0.917[0.685 ~ 1.226]	0.791[0.589 ~ 1.062]	1.172[0.837 ~ 1.64]	0.919[0.673 ~ 1.256]
History of stroke	1.166[0.786 ~ 1.73]	1.042[0.701 ~ 1.549]	1.391[0.874 ~ 2.214]	1.063[0.699 ~ 1.617]
History of coronary artery disease	0.619[0.458 ~ 0.835]	0.632[0.465 ~ 0.859]	0.604[0.431 ~ 0.846]	0.605[0.44 ~ 0.832]
History of heart failure	0.896[0.495 ~ 1.623]	0.559[0.309 ~ 1.009]	0.638[0.345 ~ 1.179]	0.646[0.354 ~ 1.178]
CKD	1.08[0.709 ~ 1.646]	1.025[0.67 ~ 1.569]	1.298[0.79 ~ 2.133]	1.293[0.815 ~ 2.051]
Beta blocker	0.962[0.731 ~ 1.265]	0.838[0.634 ~ 1.107]	1.017[0.746 ~ 1.385]	0.742[0.554 ~ 0.994]
Calcium antagonist	1.527[1.176 ~ 1.982]	1.589[1.218 ~ 2.074]	1.705[1.271 ~ 2.288]	1.411[1.066 ~ 1.868]
ACE inhibitor or ARB	1.42[1.083 ~ 1.863]	1.479[1.122 ~ 1.95]	1.273[0.938 ~ 1.728]	1.376[1.03 ~ 1.837]
Loop diuretics	0.755[0.405 ~ 1.408]	0.54[0.289 ~ 1.011]	0.454[0.24 ~ 0.861]	0.541[0.288 ~ 1.014]
Optimal dosage use	1.283[0.838 ~ 1.965]	1.285[0.836 ~ 1.976]	1.15[0.72 ~ 1.838]	1.086[0.696 ~ 1.696]

*ACE* angiotensin converting enzyme, *ARB* angiotensin receptor blocker, *BP* blood pressure, *CKD* chronic kidney disease with eGFR between 30 and 60 ml/min/1.73m2

## Discussion

The most important finding of the present study is that the prevalence of RH is 11.9 %, which was within the 95 % confidence interval of the prevalence of the previous reports [13]. As noted in that meta-analysis, as a cause of the pseudo-RH, the role of ABPM to avoid misdiagnosis was clearly demonstrated in our study [13]. As shown in our study (Table 3), the misclassification for the control status is expected in about 30 % by the presence of white-coat effect or masked uncontrolled hypertensions without using ABPM or other out-of-office BP measurement, possibly, home BP monitoring. As for the role of ABPM in RH, the misdiagnosis of RH was relatively small (6.8 %) each for white-coat effect and masked uncontrolled BP because all of the subject taking four or more drugs were classified as RH regardless of the control status. So the role od ABPM is more important when evaluating the control status which is closely related to the choice of the further therapy. Moreover once the BP was not controlled by one or two AHMs, our study showed that the response to the treatment by using three or more AHMs even including diuretics in almost all subjects was quite poor (control rate less than 31 %). Much more effective treatment modality, such as more active use of aldosterone antagonist and/or renal denervation for selected patients should be prescribed accurately for the right patient using ABPM [14, 15].

And without considering the optimal dosage criteria, the prevalence was overestimated by 1 ~ 2 % in our study which was partially consistent but the difference was not as striking as reported in the previous study [10].

In addition, our study identified the related clinical factor for the uncontrolled BP group in spite of optimal medical therapy. There was no relation suggested by Mets to uncontrolled BP group. It can be explained by the risk profile of subjects requiring three or more drugs and coronary artery disease patients too different to be compared with the most populations where Mets implied cardiovascular risk [16]. Smoking was usually known to be associated with elevation of daytime BP or masked HT. So smoking could induce overestimation of the daytime BP so that the nighttime BP should be lower than nonsmoker [17]. The relation of smoking to RH by clinic BP as shown in a previous study was not reproduced in this study probably due to small sample size [18]. The adverse effect of drinking was significant for uncontrolled BP group for nighttime and 24 h BPs. Because heavy drinking itself can be associated with RH and because nighttime BP is of prognostic importance [19], further study is needed regarding the amount of alcohol intake and the influence on the nighttime BP in RH [20]. The definition of uncontrolled BP group separately from RH seems to be better to avoid confusion but because only a minor proportion of BP in the subject treated with four or more drugs was controlled, it clinical implication needs further study.

Our study showed that CAD is consistently associated with controlled group. This finding can be potentially explained by routine use of beta blockade or RAS blockade when BP level is tolerable. Another interesting finding of our study is that heart failure is associated with daytime controlled BP group. This finding also can be explained by the standard regimen of heart failure which includes RAS blockade, beta blocker, and diuretics and mimics RH situation. Specifically, systolic heart failure patients are so sensitive to the dosage of AHM that they usually have relatively low or well controlled BP. This postulation is partly consistent with the finding that loop diuretics were more frequently prescribed in the controlled BP group. But in RH situation, heart failure itself can be aggravated by high or uncontrolled BP so that multiple drug treatment might not be the result of the standard therapy of heart failure but more likely be the attempt of controlling BP as the primary reason for heart failure.

In our study, as an independent variables in the multiple logistic regression analysis, “optimal dosage” was included so that the effect the suboptimal dosage possibly more frequently used in heart failure such as beta blocker could be adjusted. Because the proportion of the heart failure was 4.8% in our study, further study is needed for the relationship between heart failure and the RH.

The relation of AHM class to the uncontrolled BP group is very difficult to explain because our study is a cross-sectional study. The association of ARB is different from the CCB in regard to the nighttime BP. Because CCB is very potent drug to reduce BP the association can be demonstrated the perceived role of CCB in RH. But it could be a result of a secondary failure of BP control by CCB resulting in reflex sympathetic activation. Further prospective study is needed to answer if this finding is due to the pharmacological property of those drugs such as the half-life or specific effect on the nighttime BP. For the loop diuretics, it is very well known that enough diuretic therapy is very important to deal with RH. Out finding is consistent with the role of diuretics in RH. But because the use in heart failure may be a confounding factor, further study is needed.

In terms of ABPM versus home BP monitoring issues, our study showed that the nighttime BP criteria can be characterized by increased prevalence of RH and more clinical factors were related. Even though the diagnostic thresholds by ABPM based on the cardiovascular outcomes have been reported in many studies [21–25], it is reported that the nighttime BP level has the predictive power for CV events [26]. So the implications of the RH diagnosed by nighttime BP criteria should be studied in future study for the cardiovascular prognosis.

The prevalence of the masked uncontrolled HT and white-coat controlled HT or treated normalized HT in RH was quite similar to the prevalence in general hypertensive subjects [11, 27]. But as shown in our study, because most of the subject had high GCR profile, the clinical implication of masked uncontrolled HTN seems to be much more important to prevent cardiovascular events.

### Study limitations

The present study has several limitations. The generalizability can be limited because Kor-ABP registry data was driven by the tertiary referral center. But considering the RH is generally handled by the referral center, our data can be applied for referral center. And for all negative findings in this study such as the relation of MetS, CKD, or the relation of optimal dosage to the control status, it needs careful interpretation because the sample size seemed to be too small to exclude those associations. Secondly, in RH patient, it is crucial to make a differential diagnosis for secondary HT. But in our study, no standardized approach or reliable exclusion for secondary HT was performed. Thirdly, the information about the adherence as the behavioral factor which is important cause of RH was not available in the present study. Finally the relation of the clinical factors to the treatment resistance is not based on the causality because this study is a cross-sectional study.

## Conclusions

In conclusion, the prevalence itself was 11.9 % by daytime BP and it was significantly higher when using nighttime BP criteria. Control status of daytime BP was misclassified in 31 %. Smoking, drinking, coronary artery disease, calcium antagonist, and loop diuretics were associated with nighttime BP control status.
