# Erratum to: Prevalence of resistant hypertension and associated factors for blood pressure control status using Korean ambulatory blood pressure monitoring registry data

**DOI:** 10.1186/s40885-016-0048-7

**Published:** 2016-02-29

**Authors:** Sung Il Choi, Soon Kil Kim, Sungha Park, Ju Han Kim, Sang Hyun Ihm, Gwang-il Kim, Woo Shik Kim, Wook Bum Pyun, Yu-Mi Kim, Jinho Shin

**Affiliations:** Cardiology division, Department of Internal Medicine, Hanyang University, College of Medicine, 222 Wangsimni-ro Sungdong-Ku, Seoul, #133-792 South Korea; Department of Internal Medicine, Yonsei University, School of Medicine, Seoul, Korea; Department of Internal Medicine, Chonnam University, School of Medicine, GwangJu, Korea; Department of Internal Medicine, Catholic University, College of Medicine, Bucheon, Korea; Department of Internal Medicine, Seoul National University, School of Medicine, Bundang, Korea; Department of Internal Medicine, Kyung Hee University, School of Medicine, Seoul, Korea; Department of Internal Medicine, Ewha Womans University, School of Medicine, Seoul, Korea; Department of Preventive Medicine, Dong-A University College of Medicine, Busan, Korea

After the publication of the original article [1], it was brought to our attention that a number of grammatical corrections had not been implemented as requested by the author. The title has been corrected from ‘Prevalence of resistant hypertension and associated factors for blood pressure control status with optimal medical therapy using Korean ambulatory blood pressure monitoring registry data’ to ‘Prevalence of resistant hypertension and associated factors for blood pressure control status using Korean ambulatory blood pressure monitoring registry data’. All corrections have now been updated on our website.
